# Dermatology – a compulsory part of the UK medical school curriculum?

**DOI:** 10.3402/meo.v20.30212

**Published:** 2015-12-21

**Authors:** Hemal Shah, Lucia Pozo-Garcia, Marinos Koulouroudias

**Affiliations:** 1 Barts and The London School of Medicine and Dentistry, London, United Kingdom; 2 Lewisham and Greenwich NHS Trust, London, United Kingdom

**Keywords:** dermatology, OSCE, medical education, curriculum, medical school

## Abstract

Dermatological conditions form a significant number of consultations seen by general practitioners on a daily basis. There is a lack of training and formal assessment of dermatology during medical school and we propose that there should be a mandatory component in OSCEs for dermatology during medical school to enhance one's diagnostic and clinical reasoning skills which will ultimately lead to better care for the patient and efficacious use of NHS resources.

Dermatology forms an essential part of a medical student's specialty training. Inflammatory skin diseases (e.g., eczema and psoriasis) and infective diseases (e.g., impetigo and tinea) are some of the most commonly encountered conditions in primary care; it is estimated that the prevalence of skin diseases in primary care setting is around 12.4% (1, 2). Considering that there is a 50% (3) target set by the government for all medical trainees to choose a career in general practice, one would assume that there is a compulsory dermatological component in the medical school curriculum during a student's fourth, fifth, or sixth year of training; however, this is not currently the case.

There are many different forms of assessment used in medical schools throughout the United Kingdom. The most popular forms of assessment which have shown great validity in a medical student's performance as a competent and safe clinician are EMQs (extended matching questions), SBAs (single best answers), and the OSCEs (Objective Structured Clinical Examination). These forms of assessment have been widely adopted by many medical schools across the United Kingdom (4, 5). The OSCEs, in particular, form an integral component of medical students' assessment throughout their programme in most medical schools across the United Kingdom and are widely used in other healthcare professions, such as dentistry, midwifery, and optometry. OSCEs have been shown to be a crucial and reliable form of assessment in order to display a candidate's ability to carry out safe clinical examinations in patients and therefore form a key element alongside the written components (5).

Medical schools, such as Barts and The London School of Medicine and Dentistry, have their dermatology rotation in their penultimate year along with other specialties. Currently, there are dermatology examinations based on formative and summative knowledge throughout the year at Barts and The London School of Medicine and Dentistry in the form of SBAs and EMQs, but no practical clinical examination assessments , such as OSCEs. Chronic inflammatory dermatologic conditions such as eczema are very frequent in all age groups (one in five children and one in twelve adults in the United Kingdom) and can have a significant impact on social and psychological wellbeing (6). The General Medical Council in ‘*Tomorrow's Doctors*’ provides a clear framework for medical schools to design curricula. The fact that there has been a lack of practical clinical examination for dermatology considering the vast number of cases presenting in general practice on a daily basis itself is a cause for concern (7).

On the other hand, it could be argued that as most universities blueprint the quantity of questions going to be asked across all different types of examinations by the number of weeks of the specialty placement itself; it can be understandably difficult for examiners to test candidates in dermatology (8). At Barts and The London School of Medicine and Dentistry, the dermatology placement is for 2 weeks compared with placement for specialties, such as psychiatry, which is for 5 weeks and, therefore, psychiatry accounts for a major part of the assessment in both the written exams (SBAs/EMQs) and also for a significant proportion in the practical exams (OSCEs). Taking into consideration the number of dermatology cases consulted by general practitioners (12.4%) and the government target for medical trainers to choose a career in general practice, it would be greatly beneficial if there were to be a mandatory practical OSCE component in dermatology. This lack of dermatology training during medical school could be significantly costly to the NHS (National Health Service) in the future, with important dermatological ailments being missed or misdiagnosed.

We believe that practical training in dermatology should begin at an early stage in medical school, and that practical dermatological clinical examinations should be compulsory. It is paramount to implement the core skills required for a competent and confident diagnosis and management of dermatological problems leading to a better outcome for all patients and an effective use of the NHS resources (9). One of the ways in which this will be achieved is by more stringent guidelines for practical summative assessment of dermatology in the medical curricula, in which the SCOSCE (Skin Cancer Objective Structured Clinical Examination) has also recently supported the use of a practical component in evaluating a medical student's clinical performance to be effective (10). Even if this opportunity has been denied for current physicians, research has shown that postgraduate dermatology education can significantly improve the diagnostic skills of primary care physicians (11). In the long run, the early exposure to practical training could increase the efficiency and efficacy of the NHS, as it would lead to less referrals to a dermatologist (9). As Millers’ age-old pyramid of pedagogy shows, the apprentice will flourish as a clinician only if he ‘knows’ (factual knowledge) followed by ‘knows how’ (applying knowledge), ‘shows how’, and finally ‘does’ (12).
